# Career development expectations and challenges of midwives in Urban Tanzania: a preliminary study

**DOI:** 10.1186/s12912-015-0081-y

**Published:** 2015-05-08

**Authors:** Nao Tanaka, Shigeko Horiuchi, Yoko Shimpuku, Sebalda Leshabari

**Affiliations:** St. Luke’s International University, Tokyo, Japan; Muhimbili University of Health and Allied Sciences, Dar es Salaam, Tanzania

**Keywords:** Midwife, Continuing education, Tanzania, Reproductive health awareness, Life-long learning, Knowledge

## Abstract

**Background:**

Approaches to addressing the shortage of midwives are a great need especially in Sub-Saharan Africa including Tanzania. The midwifery shortage in Tanzania consists of two major causes; the first is the shortage of pre-service nursing training and the second is the low rate of retention as it is difficult to sustain midwives’ career motivations. Lack of opportunities for career development, is one of the most related problems to keep midwives motivated. Continuing education as an approach to career development can heighten midwives’ motivation and cultivate more skilled midwives who can educate other midwives or students and who could raise the status of midwives. Effective continuing education is ongoing, interactive, contextually relevant and based on needs assessment, however there is very limited research that describes Tanzanian midwives perspective of expectations for career development; hence this research is significant for revealing important and meaningful professional desires of midwives in Tanzania.

**Methods:**

This was a preliminary qualitative study, using snowball sampling to recruit 16 midwives in Tanzania. The researchers used a semi-structured interview including probing questions with both a focus group and several individuals. The data were collected from July to December 2013 and coded into categories and sub-categories.

**Results:**

There were 14 midwives in the focus group interview and two midwives in the individual interviews. Through data analysis, four major categories (with subcategories) emerged: (1) motivation for learning (to achieve the MDGs, and to raise reproductive health), (2) knowledge is power (to provide good practice based on knowledge, to be a role model, knowledge gives higher position and courage, and knowledge enables one to approach to the government), (3) there is no end to learning (hunger for learning, and ripple effect).

**Conclusions:**

From findings, four major categories plainly showed midwives’ desire for learning, however they experienced a number of barriers to access further education. Continuing education is one of the most important and effective ways to cultivate and retain midwives. In order to respond to the midwives expectations and challenges to overcome the barriers inherent in providing more continuing education, it will be necessary to increase accessible opportunities for career development in Tanzania.

## Background

Skilled birth attendants (SBAs) are midwives, nurses or doctors who have been trained to proficiency in the skills necessary to provide competent care during pregnancy and childbirth [1]. Unfortunately there is a great need globally to reduce the shortage of SBAs, especially in Sub-Saharan Africa (SSA). The probability that a woman will die from a pregnancy related cause is 1/31 in SSA, compared with 1/4300 in developed regions. Globally, around 80 % of maternal deaths are due to obstetrical complications, mainly hemorrhage, sepsis, unsafe abortion, pre-eclampsia and eclampsia, and prolonged or obstructed labour. In almost all cases these deaths are preventable when women deliver with SBAs [2]. In The United Republic of Tanzania, the shortage of SBA is a severe health service problem. The ratio of health resources in Tanzania is 0.32 professionals per 1000 populations [3], despite World Health Organization’s recent target of 2.28 professionals per 1000 populations for the SSA nations [4]. In fact, Tanzanian midwives described inadequate resources at work because there were too few staff and they were inadequately educated. There was also insufficient equipment, and too many patients in proportion to the number of midwives. Tanzanian midwives describe very difficult working condition and asserted that they need help and support in the form of being seen and acknowledged at work [5]. The midwifery shortage stems from two major causes. The first is the shortage of pre-service nursing education and teaching staff. In 2012 the number of teaching staff was 846, yet the need was 1216 in Tanzania [3]. The second is the low rate of midwife retention as it is difficult to keep them motivated. Lack of opportunities for career development as midwives is one of the most related problems to keep midwives’ motivation [3, 5].

Nils et al. [6] asserted that maintaining health worker motivation, access to training and upgrading is an important mechanism to secure motivation. According to the WHO, developing capable, motivated and supported health workers is essential for overcoming bottlenecks to achieve national and global health goals [4] and UNFPA stated that “Continuing education is essential for professional responsibility, and it is also a path to career opportunities for those who want to become educators, supervisors and researchers” [7] (p. 19). Continuing education (CE) such as in-service training, seminars, workshops and higher education (masters’ or doctoral courses) could cultivate more skilled midwives who can educate other midwives or students [6] and raise the status of midwives [8].

Willis-Shattuck et al. [9] said health worker retention was critical for health system performance, and that a key problem was how best to motivate and retain health workers in developing countries. There were seven major motivational themes: financial rewards, career development, CE, hospital infrastructure, resource ability, hospital management, and recognition/appreciation. Robertson et al. [10] reviewed the effective way that CE improves knowledge, skills, attitudes, behavior and patient health outcomes by “ongoing, interactive, contextually relevant, and based on needs assessment. (P. 153)” However, there is very limited research that describes urban Tanzanian midwives perspective of expectations for career development taking into account their career motivations; hence this research could contribute important information for the country’s efforts to scale-up the whole of midwifery.

The goal of this research is to describe the expectations and challenges of midwives for career development in Tanzania.

## Methods

### Research design and setting

This was a preliminary qualitative content analysis study implemented as part of the Asia Africa Midwifery Research Center (AMReC) for this section. That established one of the projects between Muhimbili University of Health and Allied Science (MUHAS) and St. Luke’s International University [11]. We sent a proposal and request for collaborative research to the Director of the National Institute of Medical Research, Dar es Salaam, Tanzania, and the agreement for research collaboration was obtained through AMReC.

The research field was Dar es Salaam, the largest and most developed city in Tanzania. There are four hospitals owned by the Tanzanian government [12, 13] and 13 universities and colleges in Dar es Salaam [14]; however, no universities or colleges have master’s courses of midwifery. In response to this lack, AMReC started the first collaborative project to develop the midwifery master’s course at MUHAS in Dar es Salaam, and the program commenced in 2014.

The data collection was preceded by first engaging a key informant who had come to Japan as an exchange researcher and who was also a midwife, working as a nursing administrator in Tanzania. We recruited the remaining key informants by asking to be introduced to other potential participants (snowball sampling). Inclusion criteria of participants were: (1) midwives, (2) experience working in a Tanzanian hospital, (3) bachelor’s degree in nursing or midwifery and (4) English speaking. We explained the details of the study and the interview process to those eligible. We also informed them of their right to confidentiality and to withdraw at any time without penalty. We obtained informed consent written for their participation in the interview including audio-recording. The interviews consisted of semi-structured questions with probes. The questions included such items as: How do you access higher education for career development in Tanzania now? Do you have opportunities for career development as professionals? (where and how?) Why do you want admission to the master’s course of midwifery? Why do you want access to higher education? What is your expectation for career development in Tanzania? (method, content and cost) What is your career plan as a midwife after getting a higher education?, and it took about 60 min.

Of the 16 Tanzanian midwives participating in the study 14 participated in a focus group interview, they all put in one room for only one FGD session and two participated in individual interviews, which were held in a private room. The two midwives who participated in individual interviews had a higher status of midwifery education and had study experience in a developed country. While interviewing, we took notes and audio-recorded, and then transcribe the interview for analysis. The data were collected from July to December 2013.

### Data analysis

After finishing all interviews, one of the researchers (N. T.) listened to the audio-recorded data and made transcripts then divided the transcripts into sections of 2–3 lines according to the content and then coded the lines. We grouped the codes according to conceptual relatedness and these groups were organized into subcategories and categories. Throughout the analysis, the others members (S. H. and Y. S.) checked all data and stayed immersed in the original text by going back to the transcript several times to ensure trustworthiness [15]. Finally Tanzanian midwife (L. S.) as our research member adjusted the codes and categories until we reached a consensus of meaning.

### Ethical consideration

As the research project of AMReC had already been approved by MUHAS, the approval for this particular part of research was obtained from the Research Ethics Committee of St. Luke’s College of Nursing, Japan. The approval number is 13–021.

## Results

### Participants’ background

All 16 participants were married women and had between one and five children, and their age were between the age of 28 and 56 years with a mean age of 42.6 (SD 8.88). All were midwives, including three nursing officers (nursing midwife license plus two years education). The majority (15) of the participants worked at an urban national hospital, which was one that provided the highest quality of specialized medical treatment in Tanzania, and the other was a nursing faculty in a different city. Among these 16, eight had a diploma of nursing midwifery, three had an advanced diploma of nursing midwifery, four had a bachelor’s of nursing midwifery, and one had a master’s of midwifery in Japan after getting a bachelor’s in Tanzania.

From the data analysis, four major categories were emerged: (1) Motivation for Learning, (2) Knowledge is Power, (3) There is No End to Learning and (4) Barriers to Access Higher Education (Table 1). The substance of the categories and sub-categories are presented next.

**Table 1:** Midwives expectations and challenges

Category	Subcategory
(1) Motivation for learning	a) To achieve the MDGs
	b) To raise reproductive health awareness
(2) Knowledge is power	a) To provide good practice based on knowledge
	b) To be a role model
	c) Knowledge gives higher position and courage
	d) Knowledge enables them to approach to the government
(3) There is no end to learning	a) Hunger for learning
	b) Ripple effect
(4) Barriers to access higher education	a) Lack of opportunities
	b) Financial barrier

### Motivation for learning

The first sub-category supporting Motivation for Learning was ‘to achieve the millennium development goals’. Participants expressed their opinion based on two major goals for achieving the strategic plan in Tanzania and MDGs 4 and 5, which is to reduce infant mortality and improve maternal health.

*We are motivated in order to update our knowledge for preventing maternal and neonatal mortality and morbidity. (G024)*

*We can be able to practice as midwives and achieve the Ministry of Health’s goals in that strategic plan. (G028)*

*Higher education can update our knowledge as well as skills; we can be able to meet the MDGs 4 and 5. (G032)*

The second sub-category was ‘to raise reproductive health awareness’.

*I want to give them the education that I have. In Japan, the women who lived in rural areas know danger signs, how to care for baby and management of nutrition. But in rural Tanzania some women don’t know those things. Women in Japan have education before they have their baby and there are very few young women who get in Japan. But in Tanzania, the school-age adolescents become pregnant before they reach the age of 18. They don’t have enough knowledge about babies and pregnancy. (B015)*

### Knowledge is Power

The first sub-category of Knowledge is Power is ‘to provide good practice based on knowledge’. By getting a higher education, midwives could provide skilled midwifery care more independently, positively and quickly for mothers. Midwives expressed the following ideas:

*For example, if here is the patient and I have only bachelor’s degree, I have to consult a PhD specialist. But once you are almost the same level as PhD, sometimes we can talk to patients or mothers that I hope to do more positive and respond midwifery care more quickly. (A018)*

After finishing higher education, some midwives wanted ‘to be a role model’. This was the second sub-category.

*My plan is to be a clinician as a midwife, being a mentor for other midwives even the student and others who come from other countries or from other regions. (G055)*

*I believe that maybe after getting masters degree, I will be able to guide the juniors. (A003)*

The third sub-category was ‘knowledge gives higher position and courage’.

*Our hospital is a super specialized hospital, so I want to be a specialist in midwifery and I can provide my care with high quality. (G042-043)*

*If we were able to be policy maker, the government might improve midwives salary. (A021-022)*

*I think knowledge gives you more courage. (A009)*

The fourth sub-category was ‘knowledge enables one to approach the government’. In order to improve maternal and child health (MCH) and reproductive outcomes in Tanzania, they also need government’s power, therefore they were motivated to make a contribution to society or make a policy recommendation as a policy maker or researcher.

*To me, what I know is knowledge is power. (G042)*

*I’ve already write something on my masters thesis for government. If I can have a master or PhD in midwifery, you can tell the government to support maternal and child health. So if I write thesis more, the government would notice that ‘We have to take action for mothers and children in Tanzania. (B017)*

### There is no end to learning

The first sub-category of There is No End to Learning was ‘hunger for learning’. They explicitly expressed their thought that they could achieve an advance stage by learning.

*I want to study more and more. There is no end to education. (B002-003)*

*Master’s is enough, but there is another stage I can learn more. Learning is continuing process. (B003)*

*I’m looking forward to getting a chance to access higher education for professional development. (G014)*

The second sub-category was ‘ripple effect’. Midwives had an expectation that their knowledge would be used to help women and children in Tanzania.

*There are so many regions and hospitals in Tanzania, so I cannot go everywhere. Even if I could go, teach and work at a hospital, it is not enough. To give my knowledge what I have received from higher education for many nursing students is important. (B005-008)*

### Barriers to access higher education

The first sub-category of Barriers to Access Higher Education was ‘lack of opportunities’.

*The problem is that there are many who want to go. More than five at a time, but this year only two-four could attend. (G027)*

The second sub-category was ‘financial barriers.’

*If you can get a chance for further studying you have to struggle by yourself to get sponsorship. (G002)*

## Discussion

### Motivation to improve MCH and reproductive outcomes

The category Motivation for Learning including two subcategories ‘to achieve the MDGs’ and ‘to raise reproductive health awareness,’ this is totally necessary for improving MCH and reproductive health in Tanzania. In order to achieve their goals, trained midwives who can provide quality midwifery services are needed throughout Tanzania. The other expectation they had was to maintain high quality care for patients and mothers. UNFPA [16] also stated: “Quality midwifery services are a well-documented component of success in saving the lives of women and newborns as well as promoting their health (p. 1).”

Midwives’ desire to provide sex education for young women or nursing students is linked to the National Bureau of Statistics [Tanzania] and ICF MACRO’s [17] statement; “Educational attainment has a strong effect on reproductive behavior, contraceptive use, fertility, infant and child mortality, morbidity, and attitudes and awareness related to family health and hygiene. Education provides people with the knowledge and skills that can lead to a better quality of life (p. 43).” Madeni et al. [18] conducted reproductive health awareness program for adolescents in urban Tanzania because they had limited knowledge about secondary sexual characteristics. Throughout the program, the percentage of correct answers were increased, therefore this approach was one of the effective way to increase their knowledge. In SSA, the rate of very early childbearing among adolescents is the highest among childbearing age groups and was caused by lower educational attainment and poverty and there has been little progress since 1990 [19]. Hence, midwives’ expectation to raise reproductive health awareness especially for adolescents or mothers would bring about an improvement of MCH and reproductive outcomes.

### Desire for learning

The midwives in urban Tanzania have a desire for learning and also the right for life-long learning therefore CE is especially needed as a path to career opportunities for those who want to become educators, supervisors and researchers [6]. Midwifery CE is defined in the Global Standards for Midwifery Regulation [8] as: “ongoing education undertaken from the time of the first qualification throughout one’s career to enhance or maintain a midwife’s level of competence (p. 10).”

It was clear that respondents were dissatisfied with their current status or positions as midwives, even in the urban area, and they also wanted a higher salary. Häggström et al. said, the 29 Tanzanian nurse midwives working in rural area also had workplace distress and were suffering from the lack of respect, appreciation, and influence [5]. A survey of studies of Western nurses’ (United Kingdom, United States and Canada) reasons for participating in CE were: personal certification, joy of learning, increased knowledge of new techniques and self-assurance [20]. Midwives in developed countries and the midwives in Tanzania have the same expectation; they want to access opportunities for learning more.

Of the 26,023 Tanzanian midwives, 953 live and work in English-speaking developed countries [21]. Lack of opportunity and education causes the brain drain from developing countries especially SSA to developed countries [21, 22]; African midwives living in UK noted that personal development and career development were important factors during working life and they expressed a strong ambition to develop their skills and knowledge [23]. It is clear that CE is greatly needed in order to prevent the brain drain of midwives who are motivated to learn.

### Overcome the barriers

Accessible CE is needed to reach as many midwives as possible in urban and rural Tanzania, for example, distance education, blended learning which is a combination of face-to-face and e-learning method. These methods enable midwives to continue their work in their ward or home and it is cost-effective [24, 25], and some of the most effective ways for CE to overcome the financial barriers; however there continues to be room for discussion about the viability and quality of distance education.

The Tanzania, Ministry of Health and Social Welfare [3] stated the importance for providing support to health and social welfare training institutions and recently, in-service training geared towards enabling staff to advance career and distance education is being encouraged to enable more nursing staff to enroll. Human resources for health (HRH) training in Tanzania places a great financial burden on both students and the government [17, 26]. Given the lack of finances and accessible opportunities, innovations such as six-month short programs or midwifery master’s and doctoral programs are necessary for midwifery scaling-up.

### Research limitations and perspective for the future

Although this study provided rich detail and focus regarding the perspective of midwives’ desires for career expansion there are several study limitations that must be addressed. The participants were in the minority of midwives in urban Tanzania. They mostly worked at the national hospital in urban Tanzania, therefore their work environment undoubtedly influenced their opinions or views about their expectations and challenges. Although their comments were consistent with Häggström et al. [5] broadening the base of inquiry could provide important contrasting data. Furthermore, to gain a more comprehensive perspective the interviews should be set for not only midwives but also higher positioned staff like hospital managers or midwives who works at health centers.

Increasing the number of midwives who can access CE in the near future, viability, accessibility and quality of learning modalities and teaching for distance education and e-learning, or learning subjects they want and need must be considered. This as preliminary research may helpful to consider the possibility of variety of education program or CE ways.

## Conclusions

Throughout the interviews from 16 midwifery participants, four major categories were expressed. The expectations were ‘Motivation to Learning’, ‘Knowledge is Power’, ‘There is no End to Learning’ and ‘Barriers to Access Higher Education’. These findings plainly showed their desire for learning, however they experienced a number of barriers to access higher education. CE is one of the most important and effective ways to cultivate and retain midwives. In order to respond to the midwives’ expectations and challenges to overcome the barriers inherent in providing more CE, it will be necessary to increase accessible opportunities for scaling-up as professionals in Tanzania.
